# Screening and genetic engineering of marine-derived *Aspergillus terreus* for high-efficient production of lovastatin

**DOI:** 10.1186/s12934-024-02396-z

**Published:** 2024-05-09

**Authors:** Han Na, Yao-yao Zheng, Yaoning Jia, Jingzhao Feng, Jizi Huang, Jihao Huang, Chang-Yun Wang, Guangshan Yao

**Affiliations:** 1 Key Laboratory of Marine Drugs and Key Laboratory of Evolution and Marine Biodiversity (the Ministry of Education of China), Institute of Evolution & Marine Biodiversity, School of Medicine and Pharmacy, Ocean University of China, Qingdao, 266003 China; 2 Fujian Key Laboratory on Conservation and Sustainable Utilization of Marine Biodiversity, Institute of Oceanography, Minjiang University, Fuzhou, 350108 China; 3 Laboratory for Marine Drugs and Bioproducts, Qingdao National Laboratory for Marine Science and Technology, Qingdao, 266237 China; 4 College of Life Science, Fujian Agriculture and Forestry University, Fuzhou, 350002 China; 5 School of Future Technology, Fujian Agriculture and Forestry University, Fuzhou, China

**Keywords:** Marine-derived *Aspergillus terreus*, Lovastatin, Strong promoter, LovE, Genetic engineering

## Abstract

**Background:**

Lovastatin has widespread applications thanks to its multiple pharmacological effects. Fermentation by filamentous fungi represents the major way of lovastatin production. However, the current lovastatin productivity by fungal fermentation is limited and needs to be improved.

**Results:**

In this study, the lovastatin-producing strains of *Aspergillus terreus* from marine environment were screened, and their lovastatin productions were further improved by genetic engineering. Five strains of *A*. *terreus* were isolated from various marine environments. Their secondary metabolites were profiled by metabolomics analysis using Ultra Performance Liquid Chromatography–Mass spectrometry (UPLC–MS) with Global Natural Products Social Molecular Networking (GNPS), revealing that the production of secondary metabolites was variable among different strains. Remarkably, the strain of *A*. *terreus* MJ106 could principally biosynthesize the target drug lovastatin, which was confirmed by High Performance Liquid Chromatography (HPLC) and gene expression analysis. By one-factor experiment, lactose was found to be the best carbon source for *A*. *terreus* MJ106 to produce lovastatin. To improve the lovastatin titer in *A*. *terreus* MJ106, genetic engineering was applied to this strain. Firstly, a series of strong promoters was identified by transcriptomic and green fluorescent protein reporter analysis. Then, three selected strong promoters were used to overexpress the transcription factor gene *lovE* encoding the major transactivator for *lov* gene cluster expression. The results revealed that compared to *A*. *terreus* MJ106, all *lovE* over-expression mutants exhibited significantly more production of lovastatin and higher gene expression. One of them, LovE-b19, showed the highest lovastatin productivity at a titer of 1512 mg/L, which represents the highest production level reported in *A*. *terreus*.

**Conclusion:**

Our data suggested that combination of strain screen and genetic engineering represents a powerful tool for improving the productivity of fungal secondary metabolites, which could be adopted for large-scale production of lovastatin in marine-derived *A*. *terreus*.

**Supplementary Information:**

The online version contains supplementary material available at 10.1186/s12934-024-02396-z.

## Introduction

Lovastatin, a polyketide compound produced by filamentous fungi, is the most frequently used drug for the treatment of hypercholesterolemia as a competitive inhibitor of HMG-CoA reductase [1]. In addition to lowering cholesterol, statins are also recently reported to have other attractive pharmacological effects [2]. Lovastatin displayed significant anti-tumor efficacy in a variety of cancers, such as breast, liver, cervical, lung, and colon cancers by inhibiting cell proliferation, regulating cancer cell signaling pathways, or inducing apoptosis and cell cycle arrest [2]. Andrew Robson found that lovastatin improves both cardiomyocyte and endothelial cell function in induced pluripotent stem cells derived from patients with dilated cardiomyopathy [3]. Osterweil et al. reported that lovastatin could improve excess hippocampal protein synthesis in the mouse model of fragile X syndrome and thus prevent epileptogenesis [4]. Furthermore, lovastatin and its derived drugs are reported to protect cardiomyoblasts against anthracycline-induced cardiac toxicity [5], possessing neuroprotection, anti-microbial, and anti-inflammatory activities [6]. In view of its unique physicochemical property and high biosafety, lovastatin and its derivatives could be re-purposed to finding new therapeutic usages, such as anti-cancer, anti-epileptogenesis, neuroprotection, and so on.

In nature, the capacity of lovastatin biosynthesis is confined to few species of filamentous fungi, including *Monascus* spp., *Penicillium* spp., *Aspergillus terreus*, and *Pleurotus ostreatus* [7]. Among them, *A. terreus* and *M. pilosus* represent two major producers of lovastatin [7, 8]. However, the mycotoxin citrinin is also accompanied to biosynthesis during the fermentation production of lovastatin in *Monascus* sp. [8]. Since its discovery, fermentation has been the major production way of lovastatin-derived drugs, and *Aspergillus terreus* represents the most dominant strains of lovastatin production. Therefore, research on the secondary metabolism of *A*. *terreus* is a focus shared by both pharmacists and microbiologists.

Nowadays, most industrial strains producing lovastatin were derived from the wild type strain ATCC 20542, which was firstly isolated from soil in Madrid, Spain. Genome of ATCC 20542 has been sequenced and published, and the biosynthetic pathways for lovastatin were recently revealed by biochemical and genetic analysis [9, 10]. Evidence reveals that a 64 kb cluster (*lov* cluster) of 18 genes is responsible for lovastatin biosynthesis, which contains two regulatory genes, nine biosynthetic enzyme encoding genes, two transporter encoding genes, one resistance gene, and four unknown genes [10]. Two polyketide synthases, namely, lovastatin nonaketide synthase decoded by *lovB* and lovastatin nonaketide synthase encoded by *lovF,* catalyze the formation of carbon skeletons [10]. The enoyl reductase enzyme encoded by *lovC* catalyzes the synthesis of dihydromonacolin L by forming a complex with LovB [11]. The thioesterase LovG is responsible for the release of dihydromonacolin L [12]. LovA decorates dihydromonacolin L by di-oxidation reaction to produce monacolin J, and the acyltransferase LovD subsequently transfers nonaketide from LovF to the C-8 hydroxy group of monacolin J to produce lovastatin [10].

Previously, optimization of fermentation conditions was frequently achieved and effective in the lovastatin production, including media composition, pH, temperature, etc. Among them, carbon sources of fermentation media have impactful effects on the production of lovastatin. Carbon catabolite repression (CCR) triggered by easily metabolized carbon sources suppress lovastatin biosynthesis [10], therefore, the optimum carbon resources need keep searching. When the genomic information is in hand, genetic engineering strategies have also been employed to improve the production of lovastatin as well as other secondary metabolites in the wild type of *A*. *terreus*. The transcription factor LovE, which locates within the *lov* gene cluster, functions as a specific positive regulatory factor for *lov* gene expression. Recently, it was reported that over-expression of *lovE* led to a 52.5% increase in the production of monacolin J, a precursor to lovastatin synthesis [13].

Previously, most strains showing an ability for biosynthesis of lovastatin were isolated from the soil environment, but the marine-derived *A*. *terreus* received little attention. Marine-derived microbes, including *A. terreus*, thrive in harsh environments characterized by high salinity, intense pressure, hypoxia, and limited light availability. These unique conditions force the microorganism to evolve to adapt to non-optimal condition in fermentation process [14]. All of these endurance capacities endow the marine-derived *A. terreus* more robust in the scale-up fermentation [15]. Our laboratory has long been engaged in the exploration of active metabolites from marine-derived filamentous fungi, and a variety of compounds have been identified from the marine-derived *A*. *terreus* [16–18]. In this study, secondary metabolome of five *A*. *terreus* strains from different marine environments was disclosed by LC–MS/MS and molecular network analysis using Global Natural Product Social Molecular Networking (GNPS). Among them, *A*. *terreus* MJ106 attracted our more attention because of an interesting finding that lovastatin was the most abundant secondary metabolite. Various carbon sources, including glucose, lactose, mannitol, wheat bran and corn steep, on lovastatin production of the wild type strain of MJ106 were investigated, and the essential transcription regulator gene *lovE* was over-expressed by using new constitutive promoter from translation elongation factor TEF-1a to further improve lovastatin production.

## Results

### Comparative analysis of secondary metabolite profiles of various *A*. *terreus* isolates

**Fig. 1:**
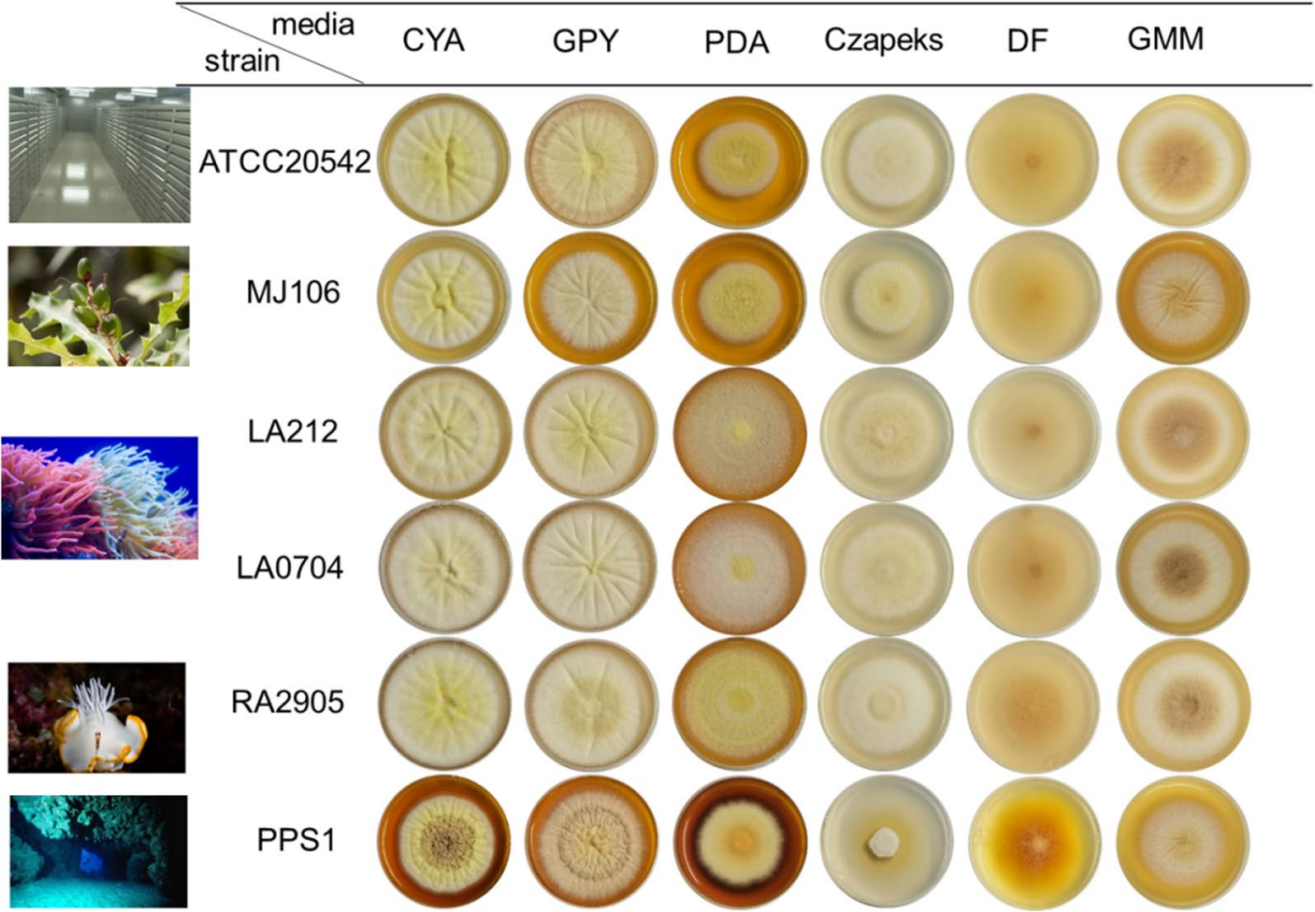
Growth of marine-derived *A*. *terreus* isolates on different media. *Aspergillus terreus* spores were inoculated on the six media plates, including CYA, GPY, PDA, LFM, TFM and GMM

During our endeavor to mine bioactive secondary metabolites of marine-derived filamentous fungi, three high-salt media, including Czapek Yeast Extract Agar (CYA), Glucose Peptone Yeast extract (GPY) and Potato Dextrose Agar (PDA) supplemented with bay salt (30 g/L), were used to recover filamentous fungi from the samples collected from submarine sediments, mangrove or marine animals. The isolation experiments for fungal strains were incubated at room temperature for 2 weeks. As a result, five *A*. *terreus* strains named LA0704, LA212, MJ106, PPS1 and RA2905 were successively isolated. For investigation of colony morphology, *A. terreus* spores were inoculated on six media plates, including Czapek Yeast Extract Agar (CYA), Glucose Peptone Yeast extract (GPY), Potato Dextrose Agar (PDA), Lovastatin Fermentation Media (LFM), Terrein Fermentation Media (TFM) and Glucose Minimal Medium (GMM), and cultured at 37 °C for 5 days. These five isolates were the same in the conidial germination, mycelial growth, and conidial development as the model strain ATCC 20542 when cultured on six different media, except for PPS1 showing slightly slower growth rate than those of other isolates (Fig. 1). Interestingly, the hyphal or conidial pigments showed apparent differences among strains grown on the same media, implying that specific secondary metabolites may occasionally be activated in each isolate.

**Fig. 2:**
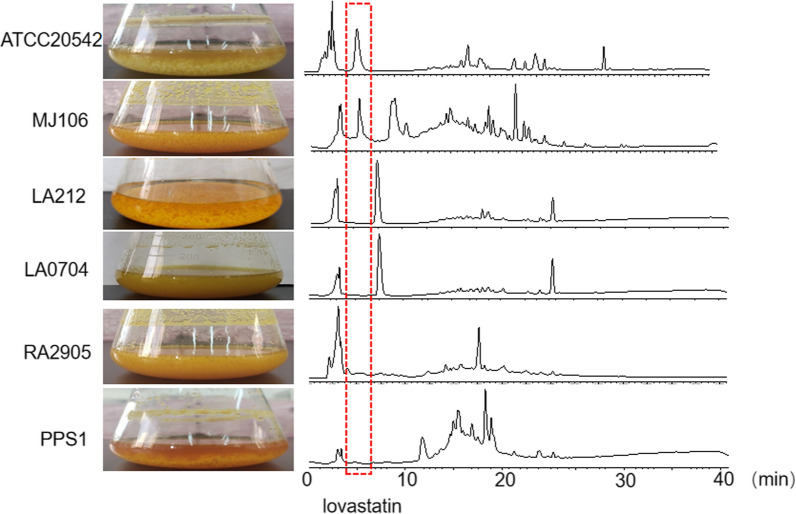
HPLC analysis of fermentation extracts of different marine-derived *A*. *terreus* isolates

**Fig. 3:**
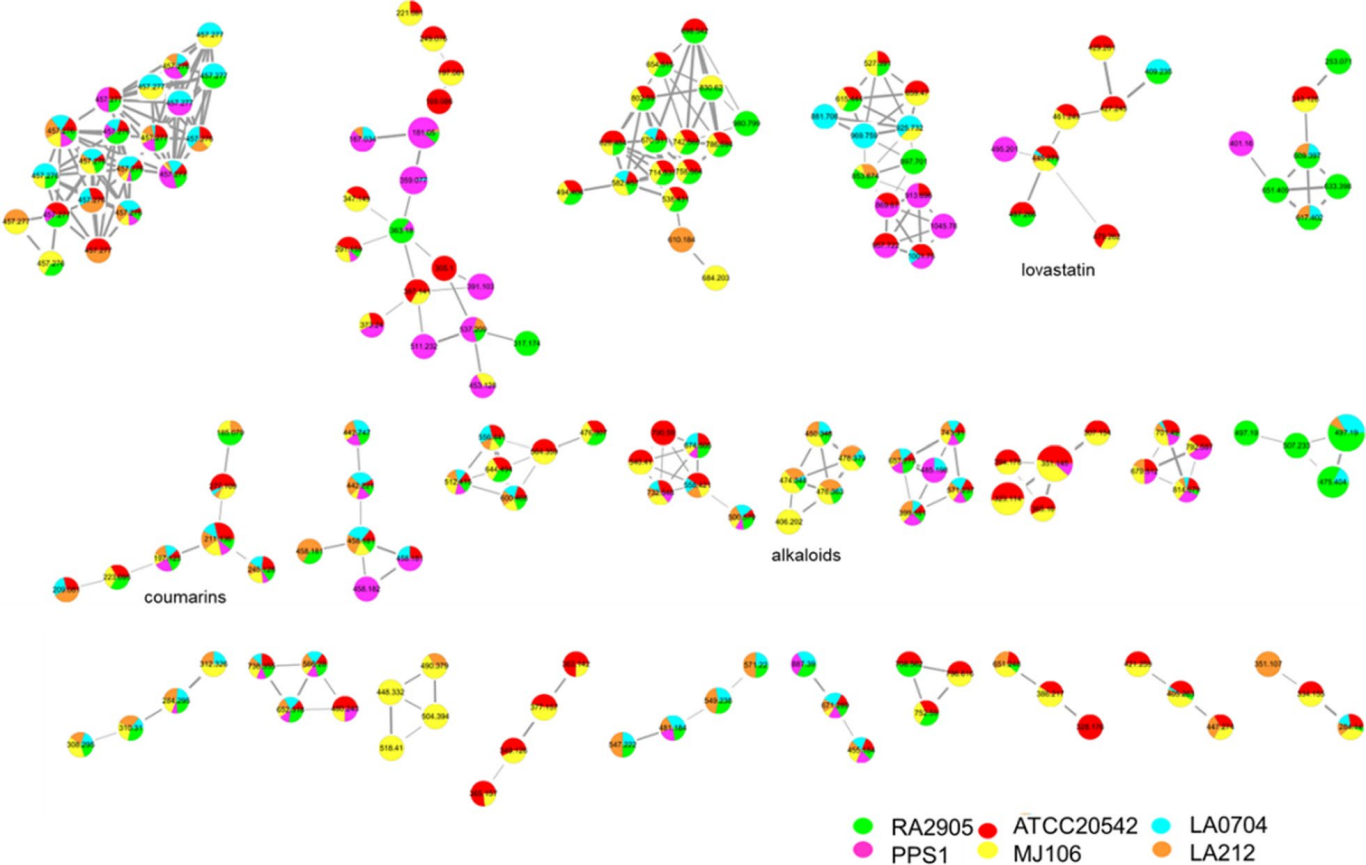
Molecular network analysis of secondary metabolites from different *A*. *terreus* isolates. Numbers represent the HRMS *m*/*z* observed for each node. Circled clusters indicate molecules positively identified

The secondary metabolite profiles of the five marine-derived *A*. *terreus* isolates and the edaphic *A*. *terreus* isolate ATCC 20542 cultured in Potato Dextrose Broth (PDB) at 30 °C, 150 rpm for 10 days were disclosed by combining HPLC and LC–MS analysis. HPLC analysis demonstrated that MJ106 exhibited similar peaks with those of ATCC 20542, and the profiles of LA0704 and LA212 were the same, while the profiles of PPS1 or RA2905 were unique, significantly different from those of other isolates (Fig. 2). The results of LC–MS and molecular network analysis (Fig. 3) further confirmed these findings.

The metabolome analysis of the five marine-derived isolates using LC–MS/MS-based molecular networking identified a total of 430 nodes (Fig. 3). Each node represents one molecular ion in the crude metabolite extract. All of these nodes were clustered, aligned, and displayed using the software of GNPS and Cytoscape 3.10.1 [19]. The results showed that each strain displayed comparative number of nodes (410–430), meaning about equal number of compounds was identified in different isolates. MJ106 and ATCC 20542 shared the majority of nodes (400/430) implying that their metabolite profiles were similar, which was consistent with the HPLC analysis. Also, most of the nodes of LA0704 and LA212 were overlapped, which coincided with their similar HPLC profiles (Figs. 2 and 3). PPS1 showed significantly more diverse chemical profile than other strains, producing most strain-specific molecular ions. Searching against GNPS database revealed that four clusters were identified to be known secondary metabolites, including lovastatins, cyclo(*D*-Trp-*L*-Pro), thiodiketopiperazines, and coumarins based on the molecular ions of 445.279, 284.14, 406.202 and 211.136, respectively (Fig. 3). These four types of metabolites represent common secondary metabolites of *A*. *terreus* strains and have been constantly identified in previous reports [18–20]. On the contrary, more than half of molecular ions in the nodes could not correlate with known metabolites from *A*. *terreus*, which warrants further purification and structure identification.

### Analysis of lovastatin production of different isolates

**Fig. 4:**
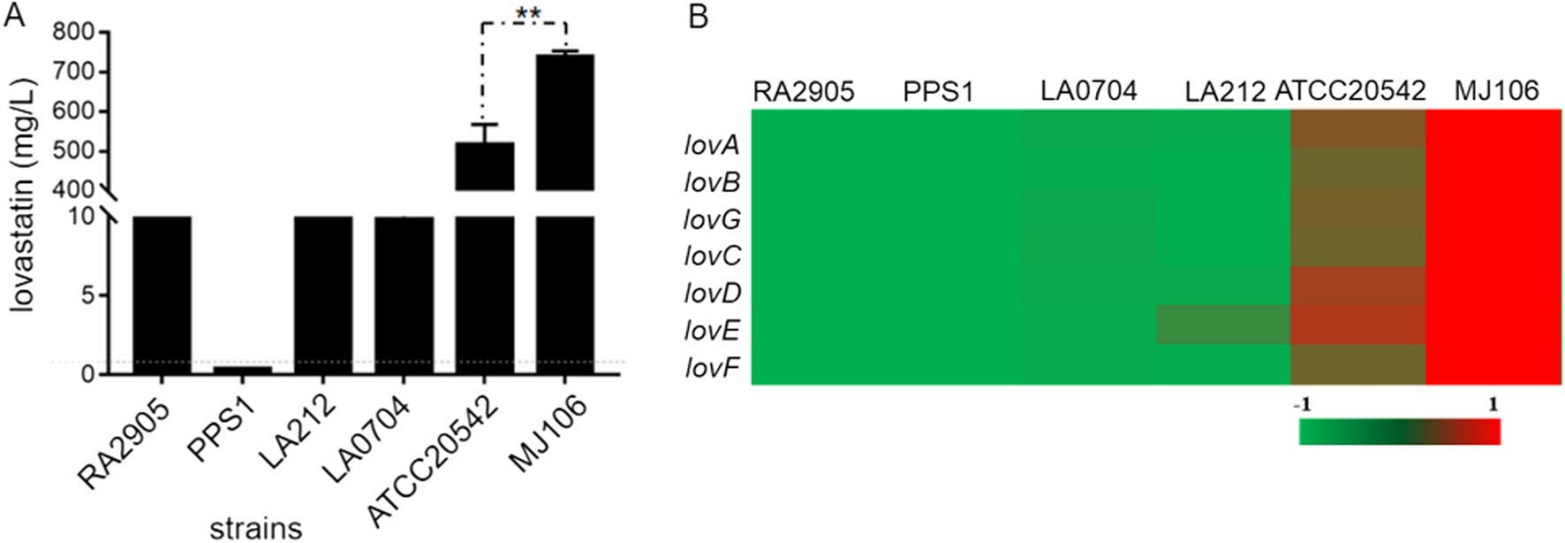
Analysis of lovastatin production for *A*. *terreus* isolates. **A** HPLC analysis of lovastatin concentrations in the culture broth when *A*. *terreus* was cultured in LFM medium. **B** qPCR analysis of *lovA-lovG* expression levels in *A*. *terreus* isolates, with the expression level of *actin* as the internal control

The above molecular network analysis suggested that the MJ106 strain was similar to the ATCC 20542 strain, producing more molecular ions and displaying a larger peak area of lovastatin compared to other isolates. It could be inferred that MJ106 may have the potential to be a producer of lovastatin like ATCC 20542. Subsequently, quantitative analysis of lovastatin titers in the fermentation of all six isolates when cultured in LFM broth was performed by LC–MS based on a standard curve. The results showed that the lovastatin titers of ATCC 20542 and MJ106 were significantly higher (10–2000-fold) than those of other *A*. *terreus* isolates (Fig. 4A), which was consistent with the above molecular network analysis. Among, *A*. *terreus* MJ106 produced the highest level of lovastatin with a titer of 740 mg/L, which was 1.5 times higher than that of the model lovastatin producer ATCC 20542. Three isolates, RA2905, LA0704 and LA212, produced approximately 10 times lower levels of lovastatin compared to ATCC 20542 or MJ106 (Fig. 4A). Interestingly, PPS1 generated a minimal titer of lovastatin in our analysis (Fig. 4A). Next, expression of lovastatin biosynthetic genes when all isolates cultured in LFM at 30 °C, 150 rpm for 48 h were determined by using real-time quantitative PCR and the results were displayed by a heatmap (Fig. 4B). Consistent with the lovastatin titer analysis, the transcript levels of seven major lovastatin synthetic genes, including *lovA*, *lovB*, *lovG*, *lovC*, *lovD*, *lovE*, and *lovF*, were significantly higher in the high-producing strains of both MJ106 and ATCC 20542, and significantly lower in those low-producing strains, including RA2905, PPS1, LA0704 and LA212. Intriguingly, the isolate MJ106 showed the highest expression for all lovastatin synthetic genes, significantly higher than ATCC 20542, suggesting MJ106 might be a potential strain for lovastatin production. Together these data indicated that the variation in production of lovastatin among these isolates was mainly attributed to discrepant gene expression of synthetic genes. Therefore, upregulation of expression levels of lovastatin biosynthetic genes might represent an efficient path to improve the titer of lovastatin.

### Screening of the optimum carbon source for lovastatin production

**Fig. 5:**
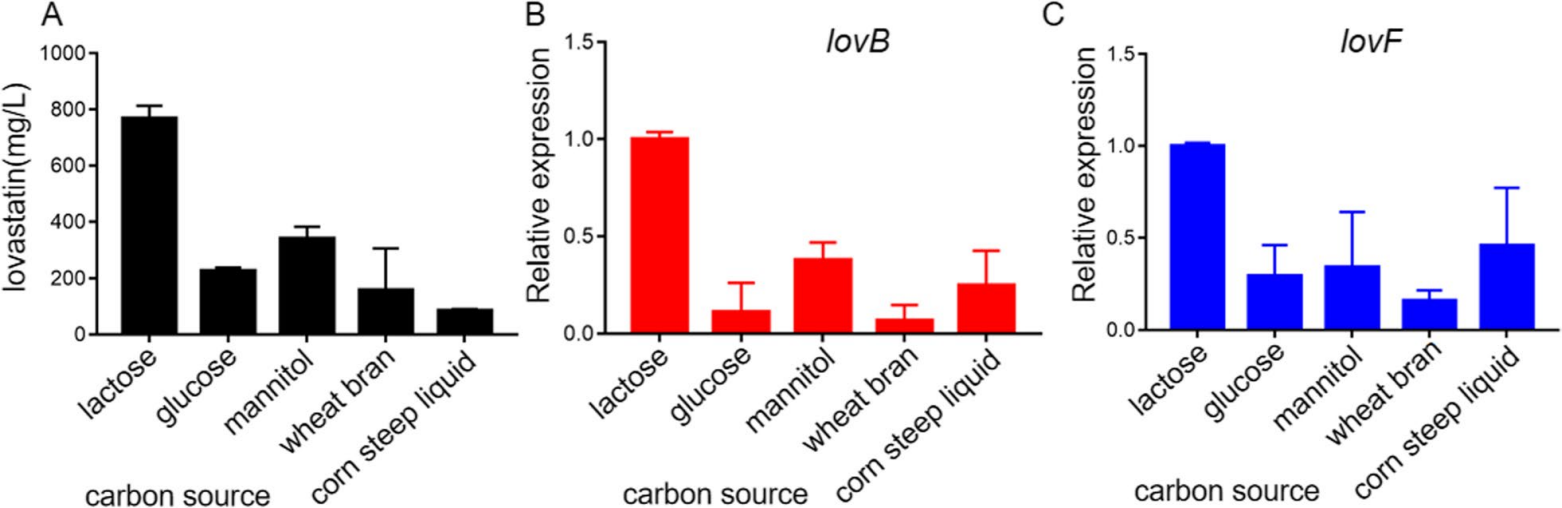
The effects of carbon sources on biosynthesis of lovastatin in *A*. *terreus* MJ106. **A** HPLC analysis of lovastatin concentrations of culture broth when *A*. *terreus* MJ106 was cultured with five different carbon sources, including lactose, glucose, mannitol, wheat bran and corn steep liquid. qPCR analysis of *lovB* (**B**) and *lovF* (**C**) expression levels in *A*. *terreus* MJ106 when cultured with five different carbon sources, with the expression level of *actin* as the internal control

Acetyl-CoA and its derivative malonyl-CoA are the building blocks of the polyketide lovastatin, which also function as major intermediates in carbon metabolism. Therefore, the carbon source has a significant impact on the production of fungal growth and biosynthesis of secondary metabolites, including lovastatin. To explore the effects of carbon sources on the lovastatin production, *A*. *terreus* MJ106 was cultured with five different carbon sources, including lactose, glucose, mannitol, wheat bran and corn steep liquid, at 30 °C, 150 rpm for 7 days. The results showed that lactose induced the highest yield, and the concentration of lovastatin reached the level of 800 mg/L. The order of optimal conditions for lovastatin production by *A*. *terreus* MJ106 was lactose > mannitol > glucose > wheat bran or corn steep liquid (Fig. 5A). Furthermore, the RT-qPCR results confirmed that the expression levels of two polyketide synthase genes, *lovB* and *lovF*, were significantly higher in lactose fermentation than those in other media (Fig. 5B, C), which is consistent with the HPLC profiles. Together, these results suggested that lactose was the optimum carbon source for fermentation of *A*. *terreus* MJ106 for lovastatin production.

### Selection of constitutive promoters and characterization using GFP reporter system

**Fig. 6:**
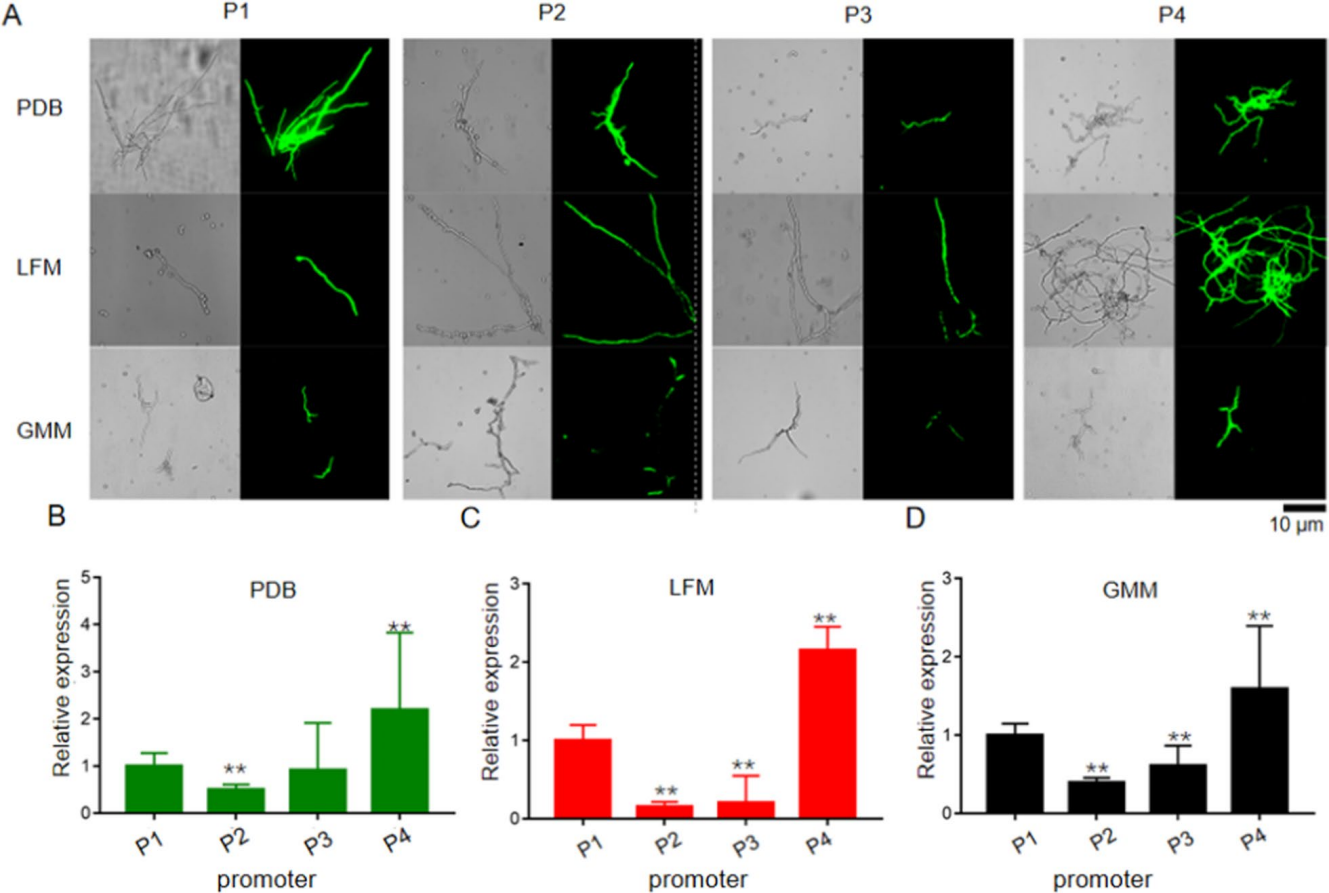
Identification of highly constitutive promoters of *A*. *terreus*. **A** Microscopic observation of GFP fluorescence intensities in the mycelia from transformants derived by four different promoters (P1–P4) under bright field image and fluorescence microscope. Scale bars represent 10 µm. **B–D** qPCR analysis of *gfp* gene in all transformants, with the expression level of *actin* as the internal control

In order to genetically engineer MJ106 for improving lovastatin production, strong promoters were identified based on transcriptome and GFP reporter analysis. As we know, both inducing and constitutive promoters have been used for gene expression in other filamentous fungi. Compared to inducing promoters, constitutive promoters enable stable expression of a variety of genes independent of external environmental factors. In our study, the top four constitutively highly expressed genes of *A*. *terreus* were identified from the transcriptome data when six *A*. *terreus* isolates were cultured in three different media, including PDB, LFM and TFM (Table 1 and Additional file 2: Table S1, Additional file 3: Table S2, Additional file 4: Table S3). Two housekeeping genes showed highest expression in each isolate (ATCC 20542, LA0704, LA212, MJ06, PPS1 or RA2905) cultured in three different media (TFM, LFM or PDB), encoding elongation factor 1-alpha and glyceraldehyde-3-phosphate dehydrogenase, respectively. The expression of genes encoding ATP synthase alpha chain or cell division cycle protein was not affected by culture composition, but showed slight differences in some isolates (Additional file 2: Table S1, Additional file 3: Table S2, Additional file 4: Table S3). To test whether the four promoters can induce gene expression in* A*. *terreus* MJ106, approximately 1 Kb of intergenic region sequences from these four genes was fused with the reporter gene *gfp* for fluorescence microscopy analysis. The microscopy results showed that cells containing all four promoter-derived *gfp* expression cassette exhibited a similar signal strength when they cultured in three different media (Fig. 6A). All transformants were cultured under three media at 30 °C, 150 rpm for 48 h, and relative transcriptional levels of *gfp* for different mutants were further determined and compared using quantitative RT-PCR analyses. The P4 promoter showed highest *gfp* expression, followed by P1, P3 and P2 in three media (Fig. 6B–D). The P1 promoter of glyceraldehyde-3-phosphate dehydrogenase has been widely used in *A*. *terreus* and other filamentous fungi [13], and the P4 from elongation factor 1-alpha has been identified in other fungi [21, 22], but not in *A*. *terreus*. Other two promoters, P2 and P3, were newly identified, and P3 showed comparable activities to the commonly used promoter P1.

**Table 1:** Promoters from constitutively highly expression genes in *A*. *terreus*

No	Gene ID	Description	Length (bp)
P1	ATEG_09817	Glyceraldehyde-3-phosphate dehydrogenase	1305
P2	ATEG_10033	Cell division cycle protein 48	1173
P3	ATEG_04767	ATP synthase alpha chain	1213
P4	ATEG_03010	Elongation factor 1-alpha	974

### Overexpressing of *lovE* to increase lovastatin using two strong constitutive promoters

Given that the low expression of biosynthetic genes is a major hindrance to lovastatin production, genetic engineering may be an efficient strategy for strain improvement, and has been widely applied in filamentous fungi to improve the production of valued secondary metabolites [23]. In the gene cluster of lovastatin, LovE encoding the Zn2Cys6 transcription factor functions as a specified and positive regulator for gene expression of other in-cluster genes [13]. Recently, the upregulation of *lovE* expression has been used to improve the production of monacolin J in *A*. *terreus*, an intermediate of lovastatin [13].

First, the gene locus of *lovE* from five marine isolates was cloned and sequenced. Interestingly, the coding sequence of *lovE* from MJ106 was the same as that from ATCC 20542, and both strains produced lovastatin at a high level. However, *lovE* from other isolates, including LA0704, LA212 and RA2905, has the same sequences, also identical to that of *A*. *terreus* NIH2624, a low lovastatin producer (Additional file 1: Fig S1). Comparison of these two *lovE* alleles revealed 61 nucleotide variations, resulting in 23 missense mutations (Additional file 1: Fig S1). Owing that the production of lovastatin in MJ106 or ATCC 20542 was higher by a wide margin than in other isolates, *lovE* from these two isolates might function as the native or authentic transcription transactivator for all *lov* gene expression.

**Fig. 7:**
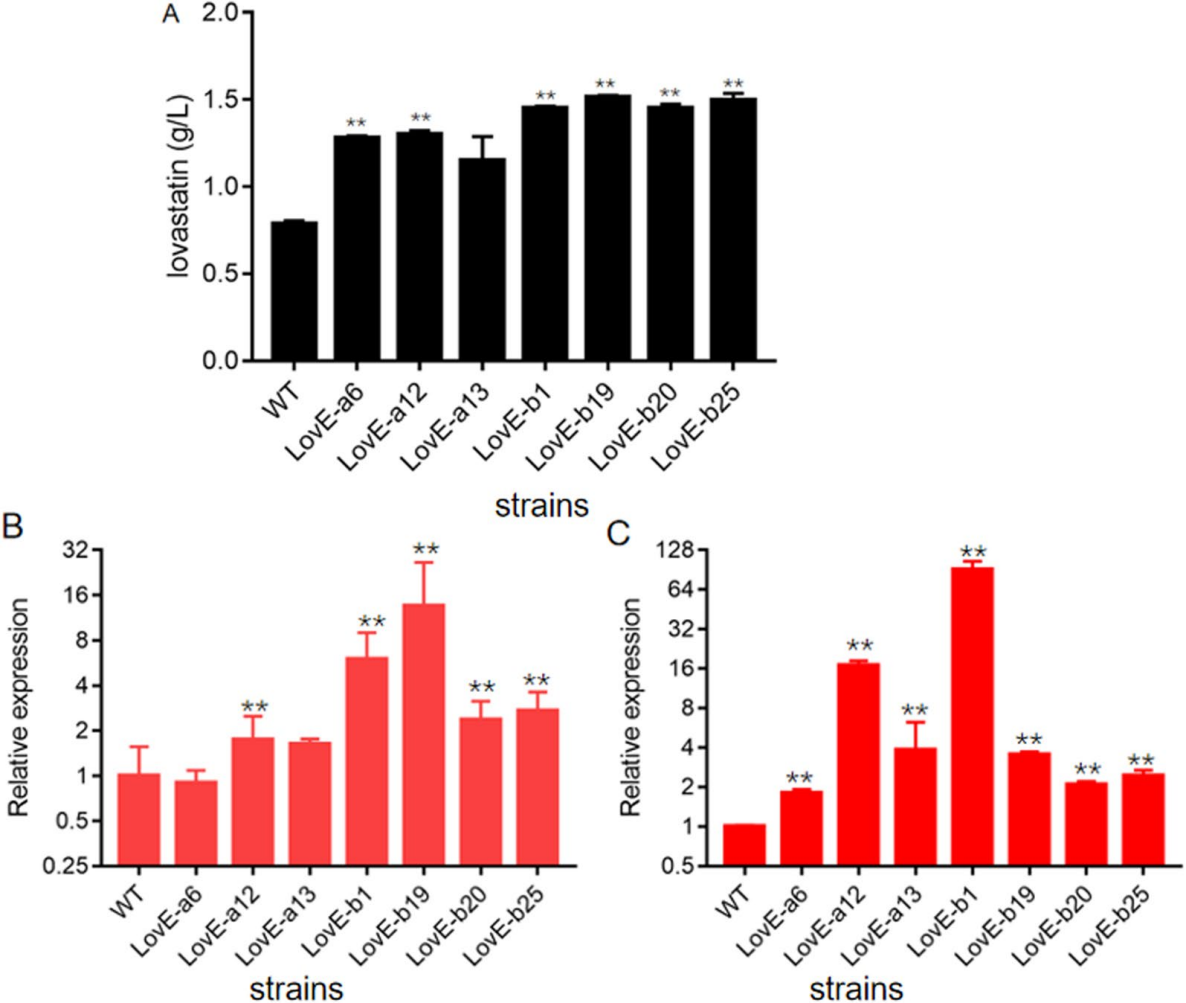
Improving lovastatin production by overexpressing the transcription activator gene *lovE* in *A*. *terreus* MJ106. **A** HPLC analysis of lovastatin concentrations of culture broth when *A*. *terreus* MJ106 and *lovE* overexpression mutants were cultured in LFM broth. **B** qPCR analysis of *lovE* expression levels in *A*. *terreus* MJ106 and *lovE* overexpression mutants when cultured in LFM media for 24 h, with the expression level of *actin* as the internal control. **C **qPCR analysis of *lovE* expression levels in *A. terreus *MJ106 and* lovE* overexpression mutants when cultured in LFM media for 48 h, with the expression level of actin as the internal control

Subsequently, the top two constitutive strong promoters, P1 and P4, were used to overexpress *lovE* gene to increase lovastatin production in *A*. *terreus* MJ106. Three and four transformants were obtained for P1 and P4, respectively. All mutants and wild type strains were fermented in LFM broth at 30 °C, 150 rpm for 10 days to investigate lovastatin production by HPLC analysis. It was found that all *lovE* overexpression mutants produce significantly higher titer of lovastatin than that of the wild type strain MJ106 (Fig. 7A). Among them, the mutant LovE-b19, whose lovE gene under the control of P4 promoter, produced the highest level of lovastatin at the titer of 1512 mg/L (Fig. 7A), representing the highest yield recorded, which was significantly higher than that of the highest production level of lovastatin reported (1342 mg/L) [24–31] (Table 2). The transcription of *lovE* in these mutants was then analyzed. The results demonstrated that the expression of *lovE* was upregulated (Fig. 7B and C), suggesting a positive correlation with the lovastatin production. Taken together, a series of high-producers of lovastatin were designed and constructed successfully by using two constitutive strong promoters in the marine-derived *A*. *terreus* MJ106.

**Table 2:** Lovastatin production reported in filamentous fungi

Strain	Method	Optimum titer (mg/L)	References
ATCC 20542	Process optimization	186.5	[24]
ATCC 20542	Process optimization	873	[25]
ATCC 20542	Process optimization	952.7	[26]
GD13	Process optimization	1342	[27]
PM3	Process optimization	240	[28]
ATCC 20542	Genetic engineering	113	[29]
ATCC 20542	Genetic engineering	152	[30]
ATCC 20542	Genetic engineering	88	[31]
MJ106	Genetic engineering	800	This study
LovE-b19	Genetic engineering and Process optimization	1512	This study

## Discussion

In this study, an outstanding lovastatin-producing *A. terreus* strain MJ106 was identified from marine environment. Lactose was determined as the optimal carbon source for this strain to produce lovastatin, which consistent with previous reports [10]. Over-expression of LovE by newly characterized strong promoters was found to be an efficient strategy in *A. terreus*.

Filamentous fungi produce a variety of secondary metabolites, which is valuable resources for drug discovery. Several lines of evidences demonstrated that there is a remarkable intraspecific variation in secondary metabolite profiles among strains from different niches [32, 33]. The isolates of *A*. *flavus* can be classified into three sub-population based on their abilities in the biosynthesis of aflatoxin and cyclopiazonic acid [34], and geographically distinct isolates of *Fusarium fujikuroi* produce distinct secondary metabolite profiles [35]. One of *A*. *flavus* strain could produce aflatoxin at a level of above 1.0 g/L [36], on the contrary, some isolates are nonaflatoxigenic [34–36]. The secondary metabolite of viridicatumtoxin can be produced in two clinical strains of MO80069 and SP260548, but not in reference strain A4 [37]. Also, our results demonstrated that the isolates of *A*. *terreus* from different environment have distinct secondary metabolite profiles. One interesting finding was that the submarine sediment-derived isolate PPS1 and *Aplysia* adnascent isolate RA2905 exhibited specific secondary metabolite profiles. Indeed, several new compounds with a variety of activities have been specifically identified from RA2905, including thiodiketopiperazine, 3,4-dihydroisocoumarin derivatives, benzyl furanones, byrones and azaphilones [16–18]. Therefore, it is proposed that continuous isolation and identification of fungal strains from various niches is helpful for fundamental research on dissection of genetic basis for intraspecific secondary metabolism, as well as for applied research on developing industrial strain for efficient bioactive compound production.

Genetic engineering represents a powerful tool to improve valuable secondary metabolite production. Strong and constitutive promoters are vital for both strain engineering and basis research. However, the available promoters for *A*. *terreus* are very limited, with only one endogenous promoter from glyceraldehyde-3-phosphate dehydrogenase gene being reported and commonly used [13]. In the present study, three constitutive promoters with strength from medium to high were identified and developed for *A*. *terreus*. Notably, the promoter of translation elongation factor 1a gene triggers significantly higher expression level of *gfp* and *lovE* than other promoters. Promoters of both glyceraldehyde-3-phosphate dehydrogenase gene and translation elongation factor 1a gene are well-characterized constitutive promoter in other fungi. Interestingly, our results suggested that the translation elongation factor 1a gene is a stronger promoter than GPDA, which was consistent with the observation in *Pichia pastoris* [21], but a reverse trend was observed in *Fusarium venenatum* [38], suggesting that the relative efficiency of these constitutive promoters is variable in distinct species.

More than half of secondary metabolite gene clusters suffer to positive regulation by pathway-specific transcription factor in filamentous fungi [38]. Thus, over-expression of the cluster transcription factors has become a straightforward and efficient tool for targeted activation or improvement of the secondary metabolite biosynthesis [38, 39]. Actually, over-expression of LovE resulted in increased *lov* gene expression and lovastatin production as well in this study. Interestingly, our results also found that nucleotide variations were identified between high-producers (MJ106 and ATCC 20542) and low-producers (RA2905, LA212 and LA0704). Surprisingly, genetic replacement of *lovE* in RA2905 by that from MJ106 did not improve its lovastatin production. The detail mechanism is under investigation. LovE is not the sole regulator for lovastatin biosynthesis in *A*. *terreus*. By identifying these unknown regulators and engineering or combined with LovE overexpression, the production of lovastatin would be further improved to higher levels.

In summary, our data suggested that developing an efficient cell factory to biosynthesize a valued secondary metabolite requires both optimization of fermentation systems and strain improvement. In addition, screening an outstanding parental strain, which is easily culturable, genetically editable, and fermentatively robust, is a fundamental requirement for cell factory construction. Our study combined these strategies resulting in a high-producing strain for lovastatin biosynthesis, reaching a level of 1.5 g/L. These findings provide a reference for microbial production of lovastatin and even other bioactive fungal secondary metabolites.

## Materials and methods

### Strains and media

*Escherichia coli* Trans1-T1 chemically competent cell (TransGen Biotech, Beijing, China) was cultured in Luria–Bertani (LB) media containing ampicillin (100 μg mL^−1^) for plasmid constructing, propagation and extraction. Five strains of *A*. *terreus* were isolated and identified from different marine habitats during 2008–2019, which were derived from the root of the mangrove medicinal plant *Acanthus ilicifolius* L. (MJ106), corals from the South China Sea (LA0704, LA212), bottom sediments from coastal area of LangQi, China (PPS1) and the fresh inner tissue of the sea hare *Aplysia pulmonica* (RA2905) [18], respectively. Fungal identification of five isolates was performed by analysis of their morphological characteristics and ITS region of the rDNA as previously described [18]. The* A*. *terreus* of ATCC 20542 was provided by Prof. Gang Liu from Shenzhen University.

These *A*. *terreus* strains and their derivative strains were grown on PDA media supplemented with or without 0.1% uracil and 0.05% uridine at 30 °C for 5–7 d to harvest conidia. To induce production of secondary metabolites, six fungal media were used, including CYA, GPY, TFM, PDB, GMM and LFM, and their compositions were listed as Additional file 5: Table S4.

### Construction of GFP reporter plasmids and *lovE* expression cassettes

All primers used in this study were listed in Additional file 6: Table S5 and synthesized by Sangon Biotech (Shanghai, China). All PCR reactions were performed using Pfu or Taq DNA polymerase from Transgen (Beijing, China). The *pyrG* gene was amplified with *A*. *fumigatus* gDNA as a template using primers pyrGF and pyrGLR. And, the *gfp* gene was amplified using primers GFP-PF and GFP-PR [40]. These two fragments were cloned into pUC-19 plasmid to generated plasmid pAT using one step cloning kit (Vazyme, Nanjing, China). Then, four selected promoters (P1–P4) were amplified with gDNA of *A*. *terreus* as a template and cloned into pAT plasmid to generate four reporter plasmids. To construct the *lovE* expression cassette, the coding region and terminator of *lovE* was amplified with primers of atlovE-R1 and atGpdA-LovE-F or atEF-LovE-F, and fused with the promoter of P1 or P4 by Double-joint PCR [41], respectively.

### Fungal transformation

To facilitate screening for positive transformants, an uracil auxotrophy parental strain of MJ106 was constructed according to our previous descriptions [15]. Preparation and DNA transformation of protoplasts of *A*. *terreus* was performed as previously described [15]. Briefly, 10^8^ fungal conidia was cultured in PDB medium supplemented with 0.1% uracil and 0.05% uridine at 37 °C for 24 h to obtain fungal hyphae. The collected hyphae were digested using an enzyme cocktail of 200 mg cellulase and 50 mg driselase. Regeneration of protoplasts was cultured on the GMM media supplemented with 1 M sucrose. All positive transformants were purified on the GMM medium and verified by PCR analysis using specific primers.

### RNA extract, cDNA synthesis and quantitative real-time-PCR

Conidia (about 10^6^/mL) of different strains was incubated and cultured in a variety of media to obtain hyphae. RNA extraction and cDNA synthesis were conducted with the Trizol agent and RNA purification Kit, according to the manufacturer’s instructions. The expression levels of target genes were quantified by qPCR using ChamQ Universal SYBR qPCR Master Mix (Vazyme Biotech, Nanjing, China). The comparative threshold cycle (CT) method was used to calculate relative gene expression levels [42] and the expression of *actin* gene was used as the internal control. All primers were listed in Additional file 6: Table S5.

### Fungal fermentation and microscopic observation

Positive transformed sporozoites were added to PDB, LFM and GMM liquid medium in equal quantities and fermented at 180 rpm, 30 °C. After 24h, the GFP fluorescence intensity of spores with the same amount of germination was captured using an Olympus BX63 fluorescence microscope equipped with an Olympus DP80 CCD camera (Olympus, https://www.getolympus.com). The excitation and emission wavelengths are 488 nm and 520 nm, respectively.

### Extraction and HPLC analysis of lovastatin

Fungal conidia (10^8^) was incubated into PDB broth for pre-culture. Hyphae was collected and transformed into fresh media for shake-flask fermentation. The culture broth from fermentation was extracted with equal amounts of ethyl acetate, and dissolved in methanol. The solvent extracts were filtrated through 0.22 disposable polytetrafluoroethylene (PTFE) prior to HPLC analysis. HPLC analysis was performed on Aligent 1260 Infinity II (USA). The extracts were separated by a C18 column of Agilent (100 × 4.6 mm i.d., 2.7 μm) and detected by UV photo diode array (PDA) at 238 nm. The regression equation for the standard solution was established for calculating the concentration of lovastatin in the fermentation broth. Three biological replicates were performed for each sample.

### LC–MS/MS and molecular networking analysis

LC–MS/MS was performed using a Waters series 2695 HPLC instrument coupled with an amaZon SL ion trap Mass spectrometer (Bruker, Karlsruhe, Germany). The HPLC conditions was as follows: column: YMC-Pack-ODS, 250 mm × 4.6 mm, 5 μm; mobile phase: (A) MeOH; (B) water with 0.1% formic acid; flow rate: 0.8 mL/min; injection volume: 10 µL; gradient: 0–40 min, 10–90% A; 40–45 min, 90–100% A; 45–50 min, 100% A; 50–60 min, 100–10% A. Mass spectra were obtained in positive ESI mode and with an automated fully dependent MS/MS scan from 100 to 2000 Da. ESI conditions were set with the capillary temperature at 320 °C and source voltage at 4.5 kV. The resulting data were converted digitally to mzXML files using Filezilla software. The molecular networking was established using the GNPS data analysis workflow using the spectral clustering algorithm [43]. The spectral networks were imported into Cytoscape 3.9.1 [19] and visualized using the force-directed layout.

### Statistical analyses

Unless otherwise indicated, experiments were performed in triplicate. Statistical analyses were performed using oRiGiNLAB software (OriginLab Corporation). P-values were determined using an unpaired two-tailed *t*-test. The statistical analyses data represent the mean value from three biological repeats; one-way ANOVA differences were considered significant when the *p* value was ≤ 0.01 (**).

### Supplementary Information

**Additional file 1: Figure S1.** Sequence alignment.

**Additional file 2: Table S1.** Highly expressed genes in LFM.

**Additional file 3: Table S2.** Highly expressed genes in PDB.

**Additional file 4: Table S3.** Highly expressed genes in TFM.

**Additional file 5: Table S4.** Media compositions.

**Additional file 6: Table S5.** Primers used in this study.

## Data Availability

The transcriptome had been deposited in GEO database (PRJNA952098) and other data generated and analyzed during this study were included in this manuscript or the additional files.
