# Supplementary figures and images for: Screening and genetic engineering of marine-derived Aspergillus terreus for high-efficient production of lovastatin
Source: Microb Cell Fact. 2024 May 9;23:134. doi: 10.1186/s12934-024-02396-z (PMC11084141; doi:10.1186/s12934-024-02396-z)

Figure S1 Sequence alignment


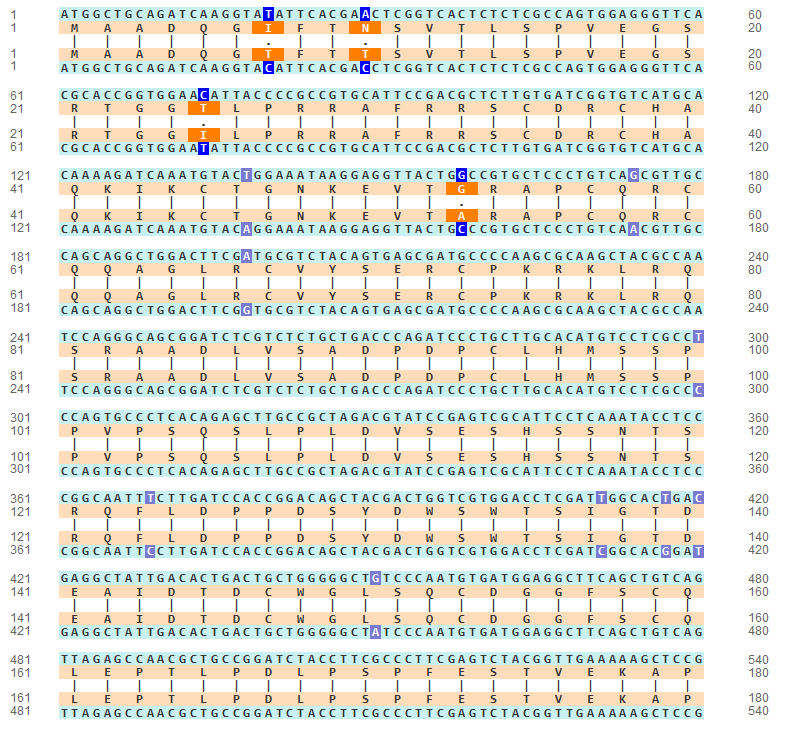


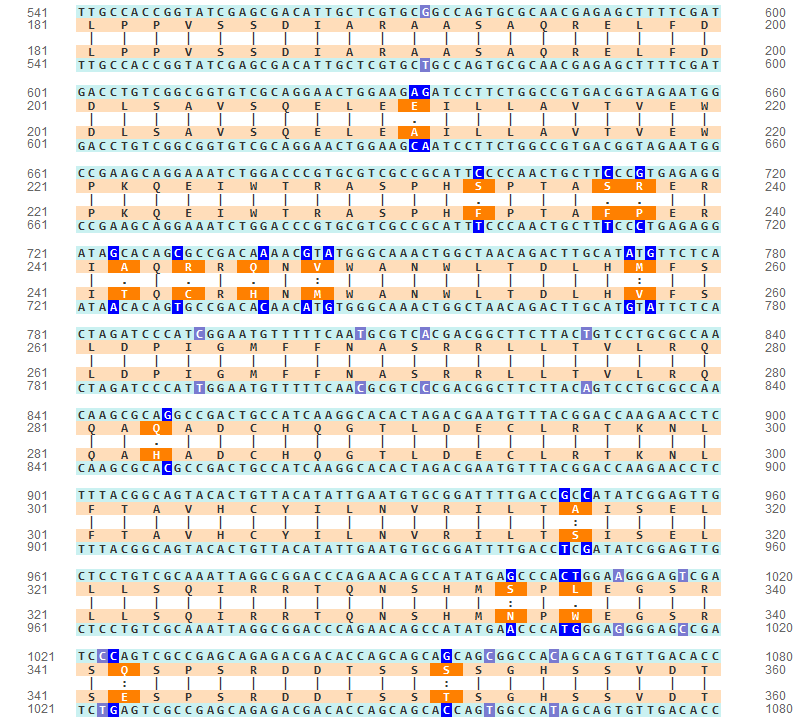


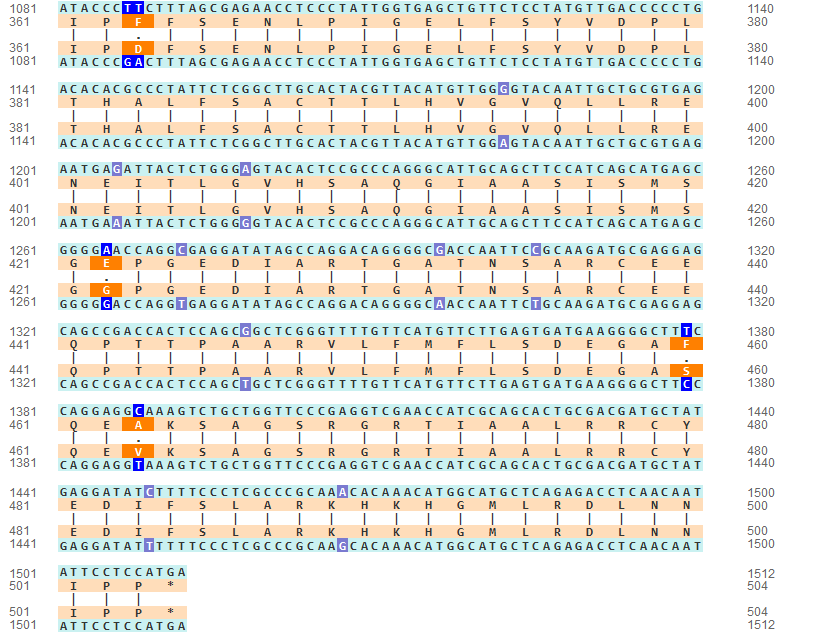


Note:

Up: MJ106 and ATCC 20542

Down: other strains.

Supplement: Supplementary file 1 — Additional file 1: Figure S1. Sequence alignment. [file 12934_2024_2396_MOESM1_ESM.docx]
