# Supplementary material for: Screening and genetic engineering of marine-derived Aspergillus terreus for high-efficient production of lovastatin
Source: Microb Cell Fact. 2024 May 9;23:134. doi: 10.1186/s12934-024-02396-z (PMC11084141; doi:10.1186/s12934-024-02396-z)
Supplement: Supplementary file 2 — Additional file 2: Table S1. Highly expressed genes in LFM. [file 12934_2024_2396_MOESM2_ESM.docx]

Table S1 Highly expressed genes of *A. terreus* and expression quantity in LFM

|  | ATCC20542 | LA0704 | LA212 | MJ06 | PPS1 | RA2905 |
| --- | --- | --- | --- | --- | --- | --- |
| ATEG_03010 | 53814 | 70217 | 137870 | 35310 | 87785 | 53007 |
| ATEG_10033 | 7321 | 9981 | 21564 | 5797 | 14022 | 11385 |
| ATEG_04767 | 27334 | 30589 | 54625 | 24029 | 43064 | 41250 |
| ATEG_09817 | 87208 | 114411 | 130206 | 112487 | 108626 | 103797 |
| actin | 2163 | 2697 | 5469 | 1951 | 3263 | 2656 |

Note:The gene expression level was measured by FPKM (Fragments per Kilobase Million), and the higher the FPKM value was, the higher the expression level was.
