# Supplementary material for: Screening and genetic engineering of marine-derived Aspergillus terreus for high-efficient production of lovastatin
Source: Microb Cell Fact. 2024 May 9;23:134. doi: 10.1186/s12934-024-02396-z (PMC11084141; doi:10.1186/s12934-024-02396-z)
Supplement: Supplementary file 3 — Additional file 3: Table S2. Highly expressed genes in PDB. [file 12934_2024_2396_MOESM3_ESM.docx]

Table S2 Highly expressed genes of *A. terreus* and expression quantity in PDB

|  | ATCC20542 | LA0704 | LA212 | MJ06 | PPS1 | RA2905 |
| --- | --- | --- | --- | --- | --- | --- |
| ATEG_03010 | 107789 | 94544 | 26927 | 109382 | 87348 | 144654 |
| ATEG_10033 | 10921 | 11061 | 16656 | 10694 | 8393 | 11129 |
| ATEG_04767 | 26224 | 23534 | 30254 | 24477 | 36793 | 52617 |
| ATEG_09817 | 18388 | 38073 | 8101 | 20965 | 56531 | 75261 |
| actin | 3062 | 3058 | 2071 | 2810 | 2924 | 3524 |

Note:The gene expression level was measured by FPKM (Fragments per Kilobase Million), and the higher the FPKM value was, the higher the expression level was.
