# Supplementary material for: Screening and genetic engineering of marine-derived Aspergillus terreus for high-efficient production of lovastatin
Source: Microb Cell Fact. 2024 May 9;23:134. doi: 10.1186/s12934-024-02396-z (PMC11084141; doi:10.1186/s12934-024-02396-z)
Supplement: Supplementary file 4 — Additional file 4: Table S3. Highly expressed genes in TFM. [file 12934_2024_2396_MOESM4_ESM.docx]

Table S3 Highly expressed genes of *A. terreus* and expression quantity in TFM

|  | ATCC20542 | LA0704 | LA212 | MJ06 | PPS1 | RA2905 |
| --- | --- | --- | --- | --- | --- | --- |
| ATEG_03010 | 24047 | 23089 | 44124 | 40578 | 18108 | 17743 |
| ATEG_10033 | 5516 | 6136 | 7911 | 7530 | 5767 | 5726 |
| ATEG_04767 | 17374 | 11873 | 14701 | 26249 | 7635 | 13060 |
| ATEG_09817 | 76794 | 91654 | 90153 | 159909 | 74911 | 41481 |
| actin | 2037 | 918 | 1219 | 3388 | 1385 | 976 |

Note:The gene expression level was measured by FPKM (Fragments per Kilobase Million), and the higher the FPKM value was, the higher the expression level was.
