# Supplementary material for: Screening and genetic engineering of marine-derived Aspergillus terreus for high-efficient production of lovastatin
Source: Microb Cell Fact. 2024 May 9;23:134. doi: 10.1186/s12934-024-02396-z (PMC11084141; doi:10.1186/s12934-024-02396-z)
Supplement: Supplementary file 5 — Additional file 5: Table S4. Media compositions. [file 12934_2024_2396_MOESM5_ESM.docx]

Table S4 Media compositions

| Culture media | Formula |
| --- | --- |
| CYA  GPY  TFM  PDB | cane sugar 3%，yeast extract 2%，NaNO_3_ 0. 3%，KCl 0.05%，FeSO_4_  0.01%，KH_2_PO_4_ 0.01 %，MgSO_4_·7H_2_O 0.05%，sea salt 3.3%  peptone 1%，yeast extract 0.5 %，glucose 2%，sea salt 3.3%  amylum 3%，peptone 0.5%,aginomoto1%，sea salt 2%  potato juice 20%, sucrose 2% , sea salt 3% |
| GMM | sucrose 2%, NaNO_3_ 0.3%, K_2_HPO_4_ 0.1%, KCl 0.05%, MgSO_4_ 0.05%, FeSO_4_ 0.01‰ |
| carbon source screening-lactose （LFM） | lactose 2%, CaCO_3_ 0.3%, yeast extract 0.5%, KCl 0.05%, KH_2_PO_4_ 0.02%, MgSO_4_ 0.15%, NaNO_3_ 0.3% |
| carbon source screening-wheat bran | wheat bran 2%, CaCO_3_ 0.3%, yeast extract 0.5%, KCl 0.05%, KH_2_PO_4_ 0.02%, MgSO_4_ 0.15%, NaNO_3_ 0.3% |
| carbon source screening-mannitol | mannitol 2%, CaCO_3_ 0.3%, yeast extract 0.5%, KCl 0.05%, KH_2_PO_4_ 0.02%, MgSO_4_ 0.15%, NaNO_3_ 0.3% |
| carbon source screening-glucose | glucose 2%, CaCO_3_ 0.3%, yeast extract 0.5%, KCl 0.05%, KH_2_PO_4_ 0.02%, MgSO_4_ 0.15%, NaNO_3_ 0.3% |
| carbon source screening-corn steep liquid | corn steep liquid 2%, CaCO_3_ 0.3%, yeast extract 0.5%, KCl 0.05%, KH_2_PO_4_ 0.02%, MgSO_4_ 0.15%, NaNO_3_ 0.3% |
