# Supplementary material for: Screening and genetic engineering of marine-derived Aspergillus terreus for high-efficient production of lovastatin
Source: Microb Cell Fact. 2024 May 9;23:134. doi: 10.1186/s12934-024-02396-z (PMC11084141; doi:10.1186/s12934-024-02396-z)
Supplement: Supplementary file 6 — Additional file 6: Table S5. Primers used in this study. [file 12934_2024_2396_MOESM6_ESM.docx]

Table S5 Primers used in this study

| Name | Sequence | |
| --- | --- | --- |
| GFP-PF | | GTAAAACGACGGCCAGTGAATTCATGGTGAGCAAGGGCGAGGA |
| GFP-PR | | ACCTGCAGGCATGCAAGCTTGGTCCAATGCATTAATGCAT |
| pyrGF | | gcctcaaacaatgctcttcaccc |
| pyrGLR | | GCCTGCAGGTCGACTCTAGAGGTCTGAGAGGAGGCACTGATG |
| P1-F | | AAACGACGGCCAGTGAATTCTTCCAGCCCTTTGAGGAGG |
| P1-R | | TCCTCGCCCTTGCTCACCATGGACTGACGCAACGACCCAG |
| P2-F | | AAACGACGGCCAGTGAATTCCAGTGCGGACAGTGGTGATG |
| P2-R | | TCCTCGCCCTTGCTCACCATTTTGGCGGTTCAAATGGATG |
| P3-F | | AAACGACGGCCAGTGAATTCGGCACGGACGTGAACTACTG |
| P3-R | | TCCTCGCCCTTGCTCACCATGGTGATGGGGGTTCAGAGGG |
| P4-F | | AAACGACGGCCAGTGAATTCACCTTGGTGGAAGCTATGTC |
| P4-R | | TCCTCGCCCTTGCTCACCATGATGGAGATAGAAGAAGGAG |
| atGpdA-LovE-F | | GACTGGGTCGTTGCGTCAGTCCATGTCCTCGCCTCCAGTGCC |
| atEF-LovE-F | | CTCCTTCTTCTATCTCCATCATGGCTGCAGATCAAGGTAC |
| atlovE-R1 | | CGCATGGGAATGAAGAAGAA |
| atlovE-R2 | | GAGTTGGCGATGTCTGATTT |
| atGPDF_1_ | | GCAGTGACGTTGGCCTGAT |
| atGPDR | | GGACTGACGCAACGACCCAG |
| atEF_1_-F_1_ | | CCAGTCGGATCAGGATTTAC |
| atEF_1_R | | GATGGAGATAGAAGAAGGAG |
| actinF | | GGTTACACCTTCTCCACCAC |
| actinR | | CCTTACGGACATCAACATCA |
| GFPRTF | | CAGTGCTTCAGCCGCTACCC |
| GFPRTR | | TTCACCTTGATGCCGTTCTT |
| lovARTF | | GGAGTCACAGCGAGTCAA |
| lovARTR | | CCTTAGAGGCAAAGAACC |
| lovBRTF | | CGGCAGATGGAGACAAAG |
| lovBRTR | | GGCGAGGACGGTTACAAT |
| lovCRTF | | GCAAATGCTACGCCTGTC |
| lovCRTR | | TCGGTCGTGACCATCTTA |
| lovDRTF | | CGCGACTCGAAGAGCAGA |
| lovDRTR | | TGGAAGAACGCAAGGGTG |
| lovERTF | | GCCCTTCGAGTCTACGGT |
| lovERTR | | TTAGCCAGTTTGCCCACA |
| lovFRTF | | GGATGGCAGGATGTGATG |
| lovFRTR | | GAAGCCCGAAAGTGAAGA |
| lovGRTF | | CCCTATCGTGGGTGCTAT |
| lovGRTR | | ATCTTTGCTGTCGTGGTC |
|  |  | |
